# The relationship between cognitive characteristics of irrational parenthood and stigma in female patients with infertility: a potential profile analysis

**DOI:** 10.1590/1980-220X-REEUSP-2024-0326en

**Published:** 2025-04-14

**Authors:** Yan Zhang, Yonghui Ding, Guangli Mi, Qiulei Li, Pin Ma, Shiling Ma, Lili Xu

**Affiliations:** 1 Ningxia Medical University, General Hospital, Yinchuan, China.; 2 Ningxia Hui Autonomous Region people’s Hospital, Yinchuan, China.; 3 Ningxia Medical University, School of Nursing, Yinchuan, China.

**Keywords:** Reproductive Health, Social Stigma, Infertility, Latent Class Analysis, Nursing, Saúde Reprodutiva, Estigma Social, Infertilidade, Análise de Classes Latentes, Enfermagem

## Abstract

**Objective:**

To explore the relationship between the potential profile of irrational parenthood cognition and stigma in female infertility patients.

**Methods:**

A cross-sectional survey was conducted among 296 infertile women using a general information questionnaire, irrational parenthood cognition questionnaire, and infertility stigma scale. A latent profile analysis was used to determine the characteristics of irrational parenthood cognition of infertile female patients, the robust three-step method was used to analyze the influencing factors of different profiles, and multiple linear regression was used to analyze the influence of the potential profile of irrational parenthood cognition on stigma.

**Results:**

Four potential profiles were identified and labeled as high irrational parenthood cognition group (19.30%), middle irrational parenthood cognition – expectation group (20.60%), middle irrational parenthood cognition – low self-esteem group (25.70%), and low irrational parenthood cognition group (34.50%). The influencing factors of the potential profile of irrational parenthood cognition in female infertility patients included ethnicity, marital status, and receiving assisted reproductive technology (*P* < 0.05). After controlling for confounding factors, the potential profile of irrational parenthood cognition was an important predictor of stigma (*P* < 0.05), which could explain 44.2% of the total variation.

**Conclusion:**

There are four potential characteristics of irrational parenthood cognition in female infertility patients, which are related to stigma.

## RESUMO

**Objetivo:**

Explorar a relação entre o perfil potencial de percepção irracional da paternidade e estigma em mulheres com infertilidade.

**Métodos:**

Foi realizada uma pesquisa transversal entre 296 mulheres inférteis, utilizando um questionário de informações gerais, um questionário de percepção irracional da paternidade e uma escala de estigma de infertilidade. Uma análise de perfil latente foi usada para determinar as características da percepção irracional da paternidade de mulheres inférteis, o método robusto de três etapas foi usado para analisar os fatores de influência de diferentes perfis, e a regressão linear múltipla foi usada para analisar a influência do perfil potencial de percepção irracional da paternidade no estigma.

**Resultados:**

Quatro perfis potenciais foram identificados e rotulados como grupo de alta percepção irracional de paternidade (19,30%), grupo expectante de percepção irracional da paternidade média (20,60%), grupo de percepção irracional da paternidade média - baixa autoestima (25,70%) e grupo de baixa percepção irracional de paternidade (34,50%). Os fatores que influenciam o perfil potencial de percepção irracional da paternidade em mulheres inférteis incluem etnia, estado civil e recebimento de tecnologia de reprodução assistida (*P* < 0,05). Após controlar os fatores de confusão, o perfil potencial de percepção irracional da paternidade foi um importante preditor do estigma (*P* < 0,05), o que poderia explicar 44,2% da variação total.

**Conclusão:**

Existem quatro características potenciais de percepção irracional da paternidade em mulheres inférteis, que estão relacionadas ao estigma.

## INTRODUCTION

Infertility was defined as the absence of contraceptive sex and non-pregnancy within 1 year^(1)^ and has become an increasingly prevalent issue in recent years. According to the World Health Organization, the global prevalence rate of infertility is 8–12%^(2)^, and infertility among women in China accounts for 16% of the childbearing-age population^(3)^.

As a concept put forward in the last 15 years, irrational parenthood cognition (IPC) refers to a cognitive bias that people have towards fertility: one must have children to achieve a happy life^(4)^. Cognition affects one’s behavioral patterns and is heterogeneous. Infertility patients believe that their inability to bear children is a physical defect, which will produce a series of stigma behaviors such as self-depreciation, social alienation, self-blame, and shame^(5, 6, 7)^. Studies have shown that pathologic shame is prevalent among women with infertility^(8)^. Pathologic shame, also known as disease shame, is a feeling of being ostracized, blamed, and not accepted caused by the disease. Disease shame can evoke negative emotions in infertile women^(9, 10)^. These negative emotions will act on the hypothalamic-pituitary- adrenal axis, leading to decreased secretion of sex hormones and impaired function of reproductive organs—a vicious circle for the treatment of infertility^(11, 12)^.

However, there are few studies on IPC and stigma^(13)^, and most of them only classify infertile women’s IPC into low and high levels based on the questionnaire scores on the IPC, without identifying the heterogeneity of characteristics of IPC in infertile women, which may lead to untargeted intervention programs developed later. Latent profile analysis (LPA), on the other hand, is centered on individuals and classifies them according to their response patterns on the scale, which has a unique advantage in determining the categories to which individuals belong and their differences^(14)^.

Therefore, this study aims to use LPA to explore the differences in the characteristics of IPC in infertile patients, and to explore the relationship between the latent categories of IPC and stigma, so as to provide a basis for the formulation of precise interventions for infertile patients.

## METHOD

### Study Design

This study used a quantitative and cross-sectional design. The study report followed the Strengthening the Reporting of Observational Studies in Epidemiology (STROBE) guidelines.

### Local

The General Hospital of Ningxia Medical University is the largest tertiary general public hospital in the Ningxia Hui Autonomous Region, responsible for the medical and health work for the local people. The Center for Reproductive Medicine was established in 2003 to treat male and female infertility. It is a modern diagnosis and treatment center integrating medical treatment, scientific research, teaching, and reproductive health popularization. It is located in the Xingqing District of Yinchuan City, with an average daily outpatient volume of about 80 people.

### Population

The population consisted of infertile women who attended the outpatient center for reproductive medicine at the General Hospital of Ningxia Medical University.

### Selection Criteria

Participants included married women of childbearing age meeting the World Health Organization’s diagnostic criteria for infertility^(15)^, with the ability to read and write independently or complete the questionnaire with the researcher assistance.

Exclusion Criteria: patients with consciousness disorder or mental disease; patients with other physical diseases, or chronic and more serious diseases.

### Sample

According to the sample size requirement of at least 5 to 10 times the number of variables, to account for potential invalid questionnaires (15–20%)^(16)^, a sample size of 108–216 was targeted, based on 18 variables. We invited 296 infertile women to participate.

### Data Collection

Infertile women attending the outpatient clinic (December 2023 – February 2024) were surveyed using the General Information Questionnaire, the Irrational Parenthood Cognitions Questionnaire, and the Infertility Stigma Scale.

#### General Information Questionnaire

The questionnaire was designed by the researcher and included demographic data (age, ethnicity, marital status, residence, educational level, occupation, per capita monthly household income, family type, length of marriage, biological children, pressure to bear children, and relationship with husband) and the disease data (type of infertility, cause of infertility, years of infertility, fertility treatment time, whether or not they have received assisted reproduction techniques, and stage of treatment).

#### Irrational Parenthood Cognitions Questionnaire (IPCQ)-14

IPCQ was developed by Fkkes and then translated into Chinese by Lvy et al.^(12)^. The scale utilized in this study consisted of 14 items, measuring women of childbearing age’s cognition of childbearing using a 5-point Likert scoring method (1‒5). The total score on the scale ranged from 14 to 70, where higher scores indicated more expected to have a child. Forty-two represented the medium level. Cronbach’s α for the scale was 0.870.

#### The Infertility Stigma Scale (ISS)-27

ISS was developed by Fu et al.^(17)^. The scale used to measure the stigma of female patients with infertility contains 4 dimensions and 27 items: self-depreciation (7 items), social withdrawal (5 items), the humiliation by the surrounding crowd (6 items), and humiliation by the family (9 items). Each item was scored on a 5-point Likert scale (1-5 points). The total score on the scale ranged from 27 to 135. Higher scores signified more stigma. Scores from 27 to 63 were categorized as low stigma, 64-100 as moderate stigma, and 101-135 as severe stigma. The Cronbach’s α for the total scale and each dimension ranged from 0.77 to 0.94.

## DATA ANALYSIS AND TREATMENT

Data were analyzed using IBM SPSS Statistics 27.0 and Mplus 8.3 software, SPSS Statistics 27.0 for statistical description, and chi-square tests for group comparisons. Logistic regression and the Kruskal-Wallis H test assessed relationships between variables. Mplus 8.3 software was used for LPA. The potential profile model fit indices are: (1) Akaike Information Criterion (AIC), Bayesian Information Criterion (BIC), and Adjusted Bayesian Information Criterion (aBIC), the smaller the values of the three indicators, the better the fit of the model. (2) Entropy represents the accuracy of classification, the value range is 0 ~ 1, the higher the Entropy value, the higher the accuracy. (3) The P-value was tested based on the Lo-Mendell Rubin Likelihood Test (LMR) and Bootstrap Likelihood Ratio Test (BLRT). *P* < 0.05 indicates that the model is significantly better than the previous model.

## ETHICAL ASPECTS

This study was approved by the Ethics Committee of the General Hospital of Ningxia Medical University (KYLL-2024-1027). All participants signed the Informed Consent Form (ICF) and voluntarily participated in this study.

## RESULTS

### General Information and Perceptions of Irrational Fertility and Stigma Scores of Survey Respondents

A total of 302 questionnaires were distributed in this survey, excluding 6 questionnaires for the same answer entry and order of answers, 296 valid questionnaires were recovered, and the effective questionnaire recovery rate was 98.01%.

A total of 296 infertile women aged 20–48 (31.34 ± 4.70) years participated in the survey, and the rest of the general information is shown in Table 1. The total score of IPC in infertile female patients ranged from 14 to 70 (38.45 ± 13.30); the total score of ISS ranged from 27 to 135 (55.37 ± 21.89), the score of the dimension of self-depreciation ranged from 7 to 35 (14.11 ± 6.40), social withdrawal dimension score 5~25 (12.78 ± 5.18), the stigma of surrounding people dimension score 9~45 (17.23 ± 7.85), and stigma of family dimension score 6~30 (11.25 ± 5.13).

**Table 1:** Univariate analysis of the latent profile of irrational parenthood cognition in female infertility patients – Yinchuan, Ningxia, China, 2023.

Variables		Overall (*n* = 296)	Profile 1 (*n* = 57)	Profile 2 (*n* = 61)	Profile 3 (*n* = 76)	Profile 4 (*n* = 102)	E.S.	*P*
Age	≤30	147 (49.7)	35 (61.4)	24 (39.3)	32 (42.1)	56 (54.9)	14.281	0.025
	31~	131 (44.3)	16 (28.1)	34 (55.7)	41 (53.9)	40 (39.2)		
	≥40	18 (6.1)	6 (10.5)	3 (4.9)	3 (3.9)	6 (5.9)		
Ethnic groups	Han ethnic group	212 (72.0)	41 (71.9)	35 (57.4)	56 (73.7)	80 (78.4)	8.579	0.035
	Other ethnic group	84 (28.0)	16 (28.1)	26 (42.6)	20 (26.3)	22 (21.6)		
Marital status	First marriage	257 (86.8)	42 (73.7)	53 (86.9)	64 (84.2)	98 (96.1)	16.693	<0.001
	Remarriage	39 (13.2)	15 (26.3)	8 (13.1)	12 (15.8)	4 (3.9)		
Residence	Rural	50 (16.9)	15 (26.3)	10 (16.4)	13 (17.1)	12 (11.8)	5.529	0.137
	Urban	246 (83.1)	42 (73.7)	51 (83.6)	63 (82.9)	90 (88.2)		
Education level	Post-secondary and below	100 (33.8)	27 (47.4)	21 (34.4)	27 (35.5)	25 (24.5)	8.738	0.033
	Bachelor’s degree or above	196 (66.2)	30 (52.6)	40 (65.6)	49 (64.5)	77 (75.5)		
Occupation	Jobless	51 (17.2)	17 (29.8)	12 (19.7)	10 (13.2)	12 (11.8)	20.246	0.063
	Migrant worker	26 (8.8)	5 (8.8)	6 (9.8)	5 (6.6)	10 (9.8)		
	Employees of enterprises and institutions	135 (45.6)	18 (31.6)	28 (45.9)	34 (44.7)	55 (53.9)		
	Individually	41 (13.9)	6 (10.5)	7 (11.5)	11 (14.5)	17 (16.7)		
	Other	43 (14.5)	11 (19.3)	8 (13.1)	16 (21.1)	8 (7.8)		
Per capita monthly household income, yuan	≤3000	36 (12.2)	10 (17.5)	6 (9.8)	9 (11.8)	11 (10.8)	13.756	0.131
	3001~	131 (44.3)	30 (52.6)	34 (55.7)	31 (40.8)	36 (35.3)		
	5001~	84 (28.4)	12 (21.1)	12 (19.7)	23 (30.3)	37 (36.3)		
	≥8000	45 (15.2)	5 (8.8)	9 (14.8)	13 (17.1)	18 (17.6)		
Family type	Backbone family	242 (81.8)	44 (77.2)	50 (82.0)	66 (86.8)	82 (80.4)	–	0.540^(1)^
	Conjugal family	6 (2.0)	0 (0.0)	1 (1.6)	2 (2.6)	3 (2.9)		
	Joint family	6 (2.0)	3 (5.3)	0 (0.0)	1 (1.3)	2 (2.0)		
	Other	42 (14.2)	10 (17.5)	10 (16.4)	7 (9.2)	15 (14.7)		
Length of marriage	≤3years	130 (43.9)	26 (45.6)	25 (41.0)	33 (43.4)	46 (45.1)	0.728	0.994
	4~7years	69 (23.3)	14 (24.6)	14 (23.0)	17 (22.4)	24 (23.5)		
	≥7years	97 (32.8)	17 (29.8)	22 (36.1)	26 (34.2)	32 (31.4)		
Biological child	No	241 (81.4)	47 (82.5)	51 (83.6)	63 (82.9)	80 (78.4)	0.945	0.815
	Yes	55 (18.6)	10 (17.5)	10 (16.4)	13 (17.1)	22 (21.6)		
Type of infertility	Primary infertility	166 (56.1)	28 (49.1)	37 (60.7)	43 (56.6)	58 (56.9)	1.672	0.645
	Secondary infertility	130 (43.9)	29 (50.9)	24 (39.3)	33 (43.4)	44 (43.1)		
Causes of infertility	Female factor	126 (42.6)	31 (54.4)	16 (26.2)	39 (51.3)	40 (39.2)	14.974	0.092
	Male factor	41 (13.9)	8 (14.0)	9 (14.8)	8 (10.5)	16 (15.7)		
	Factors on both sides	55 (18.6)	6 (10.5)	17 (27.9)	13 (17.1)	19 (18.6)		
	Unknown cause	74 (25.0)	12 (21.1)	19 (31.1)	16 (21.1)	27 (26.5)		
Years of infertility	≤1years	68 (23.0)	10 (17.5)	12 (19.7)	16 (21.1)	30 (29.4)	4.651	0.589
	2~years	126 (42.6)	25 (43.9)	26 (42.6)	36 (47.4)	39 (38.2)		
	≥5years	102 (34.5)	22 (38.6)	23 (37.7)	24 (31.6)	33 (32.4)		
Fertility treatment time	≤1years	121 (41.0)	19 (33.3)	25 (41.0)	29 (38.7)	48 (47.1)	10.599	0.102
	2~years	131 (44.4)	27 (47.4)	25 (41.0)	31 (41.3)	48 (47.1)		
	≥5years	43 (14.6)	11 (19.3)	11 (18.0)	15 (20.0)	6 (5.9)		
Assisted reproductive technology	Yes	175 (59.1)	39 (68.4)	41 (67.2)	46 (60.5)	49 (48.0)	8.938	0.030
	No	121 (40.9)	18 (31.6)	20 (32.8)	30 (39.5)	53 (52.0)		
Treatment stage	Waiting for diagnostic results	83 (28.0)	15 (26.3)	19 (31.1)	12 (15.8)	37 (36.3)	–	0.028^(1)^
	Drug/injection therapy	47 (15.9)	7 (12.3)	6 (9.8)	18 (23.7)	16 (15.7)		
	Artificial insemination therapy	9 (3.0)	0 (0.0)	2 (3.3)	4 (5.3)	3 (2.9)		
	In-Vitro Fertilization (IVF)	157 (53.0)	35 (61.4)	34 (55.7)	42 (55.3)	46 (45.1)		
Degree of fertility stress	Very large	63 (21.3)	23 (40.4)	14 (23.0)	16 (21.1)	10 (9.8)	–	<0.001^(1)^
	Relatively large	102 (34.5)	20 (35.1)	28 (45.9)	21 (27.6)	33 (32.4)		
	General	114 (38.5)	12 (21.1)	17 (27.9)	34 (44.7)	51 (50.0)		
	Relatively small	9 (3.0)	1 (1.8)	1 (1.6)	3 (3.9)	4 (3.9)		
	Very small	8 (2.7)	1 (1.8)	1 (1.6)	2 (2.6)	4 (3.9)		
Relationship with husband	Very good	183 (61.8)	33 (57.9)	37 (60.7)	45 (59.2)	68 (66.7)	–	0.261^(1)^
	Relatively good	100 (33.8)	18 (31.6)	21 (34.3)	29 (38.2)	32 (31.4)		
	General	12 (4.1)	6 (10.5)	2 (3.3)	2 (2.6)	2 (2.0)		
	Very poor	1 (0.3)	0 (0.0)	1 (1.6)	0 (0.0)	0 (0.0)		
The Infertility Stigma Scale M (P25, P75)	Self-depreciation	78.00 (62.00,96.50)	22.00 (12.00,27.00)	18.00 (12.00,21.00)	25.00 (18.00,35.00)	16.00 (13.00,19.00)	83.724	<0.001
	Social withdrawal	61.00 (51.50,71.00)	16.00 (11.50,18.50)	14.00 (11.50,18.00)	18.00 (12.00,22.00)	12.00 (7.00,15.00)	82.986	<0.001
	Humiliation by the surrounding crowd	54.00 (38.50,61.00)	13.00 (10.00,15.00)	12.50 (10.00,17.00)	17.00 (10.00,19.00)	12.00 (6.00,13.00)	90.716	<0.001
	Humiliation by the family	38.00 (30.00,54.00)	10.00 (7.00,14.00)	10.00 (6.00,12.00)	9.00 (9.00,18.00)	6.00 (6.00,12.00)	89.252	<0.001

(1) Fisher’s exact probability method. Profile 1: High IPC group; Profile 2: Middle IPC – expectation group; Profile 3: Middle IPC – low self-esteem group; Profile 4: Lower IPC group.

#### Latent Profile of Irrational Parenthood Cognition in Infertile Female Patients

**Figure 1:**
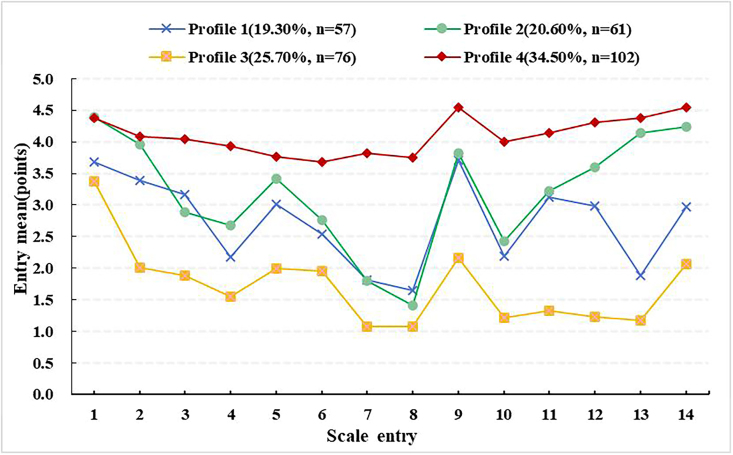
Latent profiles of irrational parenthood cognition in Female Patients with Infertility – Yinchuan, Ningxia, China, 2023.

A total of 1-5 models were fitted in this study, as shown in Table 2. Lower values of AIC, BIC, and aBIC indicate greater accuracy. The entropy value is > 0.9 for class numbers 2, 3, 4, and 5, but the LMR for classes 3 and 5 does not reach statistical significance (*P* > 0.05). The ascribing probabilities of the four latent profiles were 0.950, 0.959, 0.980, and 0.977, respectively, all of which were > 90.00%, indicating that the model of the four latent profiles was credible. Therefore, four latent profiles were selected for analysis. According to the classification results, the conditional probability diagram of latent profile was drawn, as shown in Figure 1. There were 57 cases (19.30%) in group Profile 1, and the mean score of each item was at a high level, which was named “High IPC group”. Both the Profile 2 and Profile 3 groups were located in the middle, so both were named “Moderate IPC.” There were 61 cases (20.60%) in group Profile 2, which had higher mean scores in item 1 “Having a child is the most important thing in life”, item 2 “living without a child is useless and empty”, item 13 “Not having a child is a lifelong torment” and item 14 “A person will do anything to have a child”. Therefore, it was named “Middle IPC - expectation group”; there were 76 cases in the Profile 3 group (25.70%), and this group had higher mean scores in item 3, “Some people have children easily, some people find it difficult to have children, which is a ridiculous thing”, item 7, “When you can’t have children, you feel inferior to others”, and item 8, “When you can’t have children, you start to hate your body”. Thus, it was named “Middle IPC - low self-esteem group”. There were 102 cases (34.50%) in group Profile 4, and the mean score of each item was at a low level, which was named “Lower IPC cognition group”.

**Table 2:** Fitting indicators of latent profile analysis of irrational parenthood cognition in infertile female patients – Yinchuan, Ningxia, China, 2023.

Model	AIC	BIC	aBIC	Entropy	LMR	BLRT	Class probability (%)
1 Profile	14420.301	14523.631	–	–	–	–	100
2 Profiles	13081.285	13239.970	13103.603	0.926	<0.001	<0.001	48.986/51.014
3 Profiles	12594.068	12808.109	12624.172	0.933	0.2374	<0.001	37.838/41.554/20.608
**4 Profiles**	**12424.995**	**12694.392**	**12462.885**	**0.936**	**0.0186**	**<0.001**	**25.676/20.608/34.459/19.257**
5 Profiles	12301.187	12625.939	12346.863	0.929	0.5054	<0.001	28.041/18.243/14.189/20.270/19.257

#### One-Way Analysis Variance of Latent Profile of Irrational Fertility Cognition in Female Infertile Patients

The latent profile of IPC of infertile female patients was compared in age, ethnicity, marital status, educational level, receiving assisted reproductive technology, treatment stage, and fertility pressure, and the differences were statistically significant (*P* < 0.05), as shown in Table 1.

#### Multinomial Logistic Regression Analysis of Latent Profile of Irrational Parenthood Cognition in Female Infertile Patients

The R3STEP command of the robust three-step method was used for the analysis. Multinomial logistic regression analysis was performed with the four Latent profiles as dependent variables and the variables that were statistically significant in the univariate analysis as independent variables. The dependent variables were assigned the following values: High IPC group = 1; Middle IPC – expectation group = 2; Middle IPC – low self-esteem group = 3; and lower IPC group (reference group). The independent variables were assigned as follows: age: ≤30 (1, 0), 31~(0, 1), ≥40 (0, 0); Han ethnic group = 1, other ethnic group = 2; marital status: first marriage = 1, remarriage = 2; educational level: Post-secondary and below = 1, Bachelor’s degree or above = 2; assisted reproductive technology: yes = 1, no = 2; treatment stage: waiting for diagnostic results (1, 0, 0), drug/injection therapy (0, 1, 0), artificial insemination therapy (0, 0, 1), IVF (0, 0, 0); degree of fertility stress: very large (1, 0, 0, 0), relatively large (0, 1, 0, 0), general (0, 0, 1, 0), relatively small (0, 0, 0, 1), very small (0, 0, 0, 0). The results showed that female infertile patients of Han ethnicity and in first marriages had a low IPC group, and those who received assisted reproductive technology had high and medium IPC and were expecting to have a child. See Table 3.

**Table 3:** Logistic regression analysis of the potential profile of irrational parenthood cognition in female infertility patients – Yinchuan, Ningxia, China, 2023.

Influencing factors	Options	Profile 1			Profile 2			Profile 3		
		*OR*值 (95%*CI*)	*B*	*P*值	*OR*值 (95%*CI*)	*B*	*P*值	*OR*值 (95%*CI*)	*B*	*P*值
Age	≤30	1.158 (0.307–7.508)	0.418	0.609	0.978 (0.198–4.834)	−0.022	0.978	1.380 (0.295–6.450)	0.322	0.682
	31~	0.261 (0.050–1.366)	−1.343	0.112	1.301 (0.267–6.348)	0.263	0.745	2.016 (0.434–9.351)	0.701	0.371
Ethnic groups	Han ethnic group	0.794 (0.340–1.854)	−0.230	0.595	0.358 (0.172–0.745)	−1.028	0.006	0.725 (0.349–1.505)	−0.322	0.388
Marital status	First marriage	0.053 (0.012–0.224)	−2.947	<0.001	0.275 (0.066–1.149)	−1.293	0.077	0.279 (0.076–1.023)	−1.278	0.054
Education level	Post-secondary and below	1.757 (0.761–4.057)	0.564	0.187	1.165 (0.542–2.505)	0.153	0.695	1.198 (0.588–2.438)	0.108	0.619
Assisted reproductive technology	Yes	3.396 (1.082–10.658)	1.223	0.036	3.650 (1.266–10.525)	1.295	0.017	1.733 (0.635–4.731)	0.550	0.283
Treatment stage	Waiting for diagnostic results	1.895 (0.573–6.264)	0.639	0.295	2.196 (0.731–6.596)	0.786	0.161	0.600 (0.196–1.838)	−0.051	0.371
	Drug/injection therapy	1.962 (0.478–8.053)	0.674	0.349	1.641 (0.426–6.323)	0.495	0.472	2.274 (0.716–7.221)	0.821	0.164
	Artificial insemination therapy	–	−20.043	–	1.079 (0.140–8.329)	0.076	0.942	1.942 (0.361–10.451)	0.664	0.440
Relationship with husband	Very good	10.716 (0.938–122.456)	2.372	0.056	4.920 (0.422–57.345)	1.593	0.203	3.836 (0.492–29.914)	1.344	0.200
	Relatively good	3.226 (0.303–34.630)	1.171	0.332	2.733 (0.26–28.694)	1.006	0.402	1.305 (0.187–9.130)	0.266	0.788
	General	0.743 (0.069–8.004)	−0.297	0.807	1.282 (0.121–13.595)	0.248	0.837	1.693 (0.248–11.540)	0.526	0.591
	Relatively small	1.011 (0.039–25.919)	0.011	0.995	0.984 (0.039–24.802)	−0.016	0.992	1.385 (0.122–15.747)	0.326	0.793

The independent variables were age ≥40 years, other ethnic groups, remarriage, Bachelor’s degree or above, no assisted reproductive technology, IVF, and very poor fertility stress as the reference group.

#### Relationship Between Irrational Parenthood Cognition and Pathological Stigma in Female Infertile Patients

Comparison of the total ISS scores and the scores of each dimension among infertile female patients with four latent profiles of IPC showed statistically significant differences (*P* < 0.05), as shown in Table 1. After controlling for the confounding factors of age, ethnicity, marital status, education level, acceptance of assisted reproduction technology, stage of treatment, and fertility stress, the total ISS score as the dependent variable, and the four latent profiles of IPC as the independent variable, multiple linear regression analysis was performed. The results showed that high IPC and middle IPC – expectation and middle IPC - low self-esteem were all positively predictive of stigma compared to lower IPC (all *P*<0.05), explaining 44.2% of the total variance. See Table 4.

**Table 4:** Multiple linear regression analysis of the perceived latent profile of irrational parenthood and stigma in female infertility patients – Yinchuan, Ningxia, China, 2023.

Profile	*B*	*SE*	*t*	*P*	95%*CI*
Constant	57.445	9.418	6.099	<0.001	38.906~75.983
High irrational parenthood cognition group	34.628	2.953	11.727	<0.001	28.816~40.440
Middle irrational parenthood cognition – expectation group	16.256	2.769	5.870	<0.001	10.805~21.707
Middle irrational parenthood cognition – low self-esteem group	10.249	2.528	4.054	<0.001	5.273~15.226

*R* ^2^ = 0.461, adjusted *R* ^2^ = 0.442, F = 24.405, *P* < 0.001.

## DISCUSSION

### Heterogeneity Exists in the Irrational Parenthood Cognition of Infertile Female Patients

Infertile women exhibit heterogeneity in their IPC. Using LPA, we identified 4 distinct profiles of IPC among infertile women. The higher IPC group (Profile 1), the middle IPC - expectation group (Profile 2), the middle IPC - low self-esteem group (Profile 3), and the lower IPC group (Profile 4). The higher IPC group (Profile 1) included 57 cases (19.30%), indicating a strong desire to achieve a happy life through childbearing. It suggests that healthcare professionals should transfer patients’ fertility intentions and correctly guide fertility concepts to avoid excessive IPC during the treatment of infertility. Profiles 2 and 3, accounting for 46.30% of the sample, exhibited medium levels of IPC, but differed in their underlying characteristics. Therefore, the present study used LPA to identify the heterogeneity of IPC in such patients. Early identification and tailored cognitive interventions are crucial for individuals in Profile 1. Profile 4, the largest group (34.50%), exhibited lower IPC, suggesting a need to explore intrinsic motivations for fertility and improve their cognitive outlook.

### Analysis of Influencing Factors of A Latent Profile of Irrational Parenthood Cognition in Infertile Female Patients

The results of this study showed that IPC was lower in first-married female patients with infertility compared to remarriage (*OR* = 0.053, *P* < 0.001). Marital status influences fertility intentions, with first-married individuals exhibiting lower levels of IPC^(18)^. First-married patients have just entered marriage and have a stable marital status, so they have lower willingness to have children and lower IPC. Therefore, for first-married patients, infertility should be actively treated, scientific fertility concepts should be established, and IPC should be improved. In addition, this study also found that, compared with ethnic minorities, Han ethnicity female patients with infertility had lower IPC (*OR* = 0.358, *P* = 0.006), which was similar to Shreffler’s results^(19)^. Fertility intention itself is significantly culture-specific, and ethnic differences are an important aspect of it^(20)^. Ningxia belongs to the ethnic minority areas, and due to traditional cultural thinking, ethnic minorities have earlier marriages and earlier pregnancies, while Han ethnicity has later marriages and late childbearing, which results in a lower IPC. Therefore, traditional fertility ideas should be changed to correct IPC bias in response to cultural differences.

The results of this study showed that female patients with infertility who received assisted reproductive technology had higher (*OR* = 3.396, *P* = 0.036) or moderate (*OR* = 3.650, *P* = 0.017) IPC compared to those who did not receive assisted reproductive technology, similar to the results of a foreign study^(21)^. The demanding nature of assisted reproductive technology, including invasive procedures, high costs, and emotional stress, may contribute to increased IPC. Infertility as a stigmatizing and isolating experience is a chronic stressor^(22)^. During assisted conception, long and grueling treatments, high medical costs, invasive procedures, pressures from family and society, and especially the failure of assisted conception can cause varying degrees of physiological and psychological stress in infertile patients, which can result in higher and moderate IPC. Therefore, we should focus on the IPC of infertility patients with assisted reproductive technology, actively guide the correct concept of fertility cognition, popularize the knowledge of rational fertility cognition view, and improve the level of IPC.

### Relationship Between Cognitive Characteristics of Irrational Parenthood and Stigma in Infertile Women

The results of this study showed that the higher IPC group had the highest scores in the four dimensions of infertility stigma, self-depreciation, social withdrawal, humiliation by the surrounding crowd, and humiliation by the family, followed by the middle IPC - expectation group, the middle IPC - low self-esteem group, and lastly, the lower IPC group. Higher IPC is associated with increased desire for a child, self-blame, and dissatisfaction with infertility. Higher IPC is associated with increased fear of social judgment, avoidance of child-related topics, and heightened sensitivity to child-related stimuli. The middle IPC – expectation group and the middle IPC – low self-esteem group have the middle-level scores of the four dimensions, which indicates that their desire to have a child is at a medium level. The two profiles’ apparent desire to have a child was medium, but their underlying characteristics differed. In contrast, the low IPC group scored the weakest of the 4 dimensions, indicating that the patients were not particularly looking forward to having a child. As a result, there is also no concern about what they and others think, and there are no series of psychological changes such as feelings of shame.

In addition, this study also found that after controlling for confounders, the higher IPC, the middle IPC – expectation, and the middle IPC – low self-esteem were positively predictive of disease stigma compared to lower IPC (all *P* < 0.05). Disease shame, or stigma, involves feelings of difference, rejection, and unacceptance due to illness, which can lead to stigmatization and self-depreciation during infertility events, and is closely related to fertility stress in infertility patients^(23)^. Stigma, as a negative emotion, can lead to disruption of sex hormone secretion, which can affect fertility and reduce the success rate of assisted conception^(24)^. IPC, particularly high expectancy, and low self-esteem can exacerbate feelings of sickness and shame, leading to increased fertility stress^(25)^. In the clinic, IPC can be used as an entry point for intervention, and through psychological interventions such as positive thinking therapy, group cognitive therapy, and rational emotive behavioral therapy, we can help patients replace irrational thinking styles and beliefs with rational thinking styles and beliefs, to reduce the occurrence of disease shame.

## CONCLUSION

In this study, the IPC of female patients with infertility was categorized into four profiles by LPA: High IPC group, Middle IPC – expectation group, Middle IPC – low self- esteem group, and lower IPC group. Significant characteristics existed among the latent profile and were influenced by marital status, ethnicity, and whether or not assisted reproductive technology was a factor. In addition, this study now shows that IPC traits in infertile women can positively predict stigma. Therefore, healthcare workers should identify the characteristics of different categories of patients at an early stage, use IPC as an entry point for intervention, and actively mobilize all aspects of social support to establish a correct view of parenthood cognition in patients and reduce the level of stigma in infertility patients.
