# Supplementary material for: The relationship between cognitive characteristics of irrational parenthood and stigma in female patients with infertility: a potential profile analysis
Source: Rev Esc Enferm USP. 2025 Apr 14;59:e20240326. doi: 10.1590/1980-220X-REEUSP-2024-0326en (PMC11999534; doi:10.1590/1980-220X-REEUSP-2024-0326en)
Supplement: Translation

# Translation: La relación entre las características cognitivas de la paternidad irracional y el estigma en las pacientes con infertilidad: un análisis de perfil potencial

**DOI:** 10.1590/1980-220X-REEUSP-2024-0326es

**Authors:** Yan Zhang, Yonghui Ding, Guangli Mi, Qiulei Li, Pin Ma, Shiling Ma, Lili Xu

**Affiliations:** 1 Ningxia Medical University, General Hospital, Yinchuan, China; 2 Ningxia Hui Autonomous Region people’s Hospital, Yinchuan, China; 3 Ningxia Medical University, School of Nursing, Yinchuan, China

**Keywords:** Salud Reproductiva, Estigma Social, Infertilidad, Análisis de Clases Latentes, Enfermería

## Abstract

**Objetivo:**

Explorar la relación entre el perfil potencial de percepción irracional de la paternidad y estigma en las pacientes con infertilidad.

**Métodos:**

Se realizó una encuesta transversal entre 296 mujeres infértiles utilizando un cuestionario de información general, un cuestionario de percepción irracional de la paternidad y una escala de estigma de infertilidad. Se utilizó un análisis de perfil latente para determinar las características de la percepción irracional de la paternidad de las pacientes infértiles, se utilizó el método robusto de tres pasos para analizar los factores que influyen en diferentes perfiles y se utilizó regresión lineal múltiple para analizar la influencia del perfil potencial de la percepción irracional de la paternidad sobre el estigma.

**Resultados:**

Se identificaron y etiquetaron cuatro perfiles potenciales: grupo de percepción irracional de paternidad alta (19,30%), grupo de percepción irracional de paternidad media - grupo expectante (20,60%), grupo de percepción irracional de paternidad media - autoestima baja (25,70%) y grupo de percepción de paternidad irracional baja (34,50%). Los factores que influyen en el perfil potencial de la percepción irracional de la paternidad en las pacientes con infertilidad incluyeron el origen étnico, el estado civil y la recepción de tecnología de reproducción asistida (*P* < 0,05). Después de controlar los factores de confusión, el perfil potencial de la percepción irracional de la paternidad fue un predictor importante del estigma (*P* < 0,05), lo que podría explicar el 44,2% de la variación total.

**Conclusión:**

Hay cuatro características potenciales de la cognición de paternidad irracional en las pacientes con infertilidad, que están relacionadas con el estigma.

## INTRODUCCIÓN

La infertilidad se definió como la ausencia de embarazo con relaciones sexuales sin anticonceptivos en el plazo de un año^(1)^ y se ha convertido en un problema cada vez más frecuente en los últimos años. Según la Organización Mundial de la Salud, la tasa de prevalencia mundial de la infertilidad es del 8–12%^(2)^, y la infertilidad entre las mujeres en China representa el 16% de la población en edad fértil^(3)^.

Como concepto planteado en los últimos 15 años, la cognición irracional de paternidad (CIP) se refiere a un sesgo cognitivo que las personas tienen hacia la fertilidad: uno debe tener hijos para lograr una vida feliz^(4)^. La cognición afecta a los patrones de comportamiento de una persona y es heterogénea. Los pacientes con infertilidad creen que su incapacidad para tener hijos es un defecto físico, lo que producirá una serie de comportamientos estigmatizantes como el autodesprecio, la alienación social, la autoculpa y la vergüenza^(5, 6, 7)^. Los estudios han demostrado que la vergüenza patológica es frecuente entre las mujeres con infertilidad^(8)^. La vergüenza patológica, también conocida como vergüenza por enfermedad, es un sentimiento de ostracismo, culpa y no aceptación causado por la enfermedad. La vergüenza por enfermedad puede evocar emociones negativas en las mujeres infértiles^(9, 10)^. Estas emociones negativas actuarán sobre el eje hipotalámico-hipofisario-adrenal, lo que provocará una disminución de la secreción de hormonas sexuales y un deterioro de la función de los órganos reproductivos, un círculo vicioso para el tratamiento de la infertilidad^(11, 12)^.

Sin embargo, existen pocos estudios sobre la CIP y el estigma^(13)^, y la mayoría de ellos solo clasifican la CIP de las mujeres infértiles en niveles bajos y altos basándose en las puntuaciones del cuestionario sobre la CIP, sin identificar la heterogeneidad de las características de la CIP en las mujeres infértiles, lo que puede conducir a programas de intervención no específicos desarrollados posteriormente. El análisis de perfiles latentes (LPA en la sigla en inglés Latent Profile Analysis), por otro lado, se centra en los individuos y los clasifica según sus patrones de respuesta en la escala, lo que tiene una ventaja única para determinar las categorías a las que pertenecen los individuos y sus diferencias^(14)^.

Por lo tanto, este estudio tiene como objetivo utilizar el LPA para explorar las diferencias en las características dla CIP en pacientes infértiles, y explorar la relación entre las categorías latentes de la CIP y el estigma, a fin de proporcionar una base para la formulación de intervenciones precisas para pacientes infértiles.

## MÉTODO

### Diseño del Estudio

Este estudio utilizó un diseño cuantitativo y transversal. El informe del estudio siguió las directrices del Strengthening the Reporting of Observational Studies in Epidemiology (STROBE).

### Local

El Hospital General de la Universidad Médica de Ningxia es el hospital público general terciario más grande de la Región Autónoma de Ningxia Hui, responsable del trabajo médico y sanitario para la población local. El Centro de Medicina Reproductiva se estableció en 2003 para tratar la infertilidad masculina y femenina. Es un moderno centro de diagnóstico y tratamiento que integra tratamiento médico, investigación científica, enseñanza y divulgación de la salud reproductiva. Está situado en el distrito de Xingqing de la ciudad de Yinchuan, con un volumen medio diario de pacientes ambulatorios de unas 80 personas.

### Población

La población estaba formada por mujeres infértiles que acudían al centro ambulatorio de medicina reproductiva del Hospital General de la Universidad Médica de Ningxia.

### Criterios de Selección

Entre las participantes se encontraban mujeres casadas en edad fértil que cumplían los criterios diagnósticos de infertilidad de la Organización Mundial de la Salud^(15)^, con capacidad para leer y escribir de forma independiente o completar el cuestionario con la ayuda del investigador.

Criterios de exclusión: pacientes con trastorno de la conciencia o enfermedad mental; pacientes con otras enfermedades físicas o enfermedades crónicas y más graves.

### Muestra

De acuerdo con el requisito de tamaño de la muestra de al menos 5 a 10 veces el número de variables, para tener en cuenta los posibles cuestionarios no válidos (15–20%)^(16)^, se fijó como objetivo un tamaño de muestra de 108-216, basado en 18 variables. Invitamos a participar a 296 mujeres infértiles.

### Recogida de Datos

Se encuestó a mujeres infértiles que acudían a la clínica ambulatoria (diciembre de 2023 - febrero de 2024) utilizando el Cuestionario de Información General, el Cuestionario de Cognición Irracional de Paternidad y la Escala de Estigma de la Infertilidad.

#### Cuestionario de Información General

El cuestionario fue diseñado por el investigador e incluía datos demográficos (edad, etnia, estado civil, residencia, nivel educativo, ocupación, ingresos mensuales per cápita del hogar, tipo de familia, duración del matrimonio, hijos biológicos, presión para tener hijos y relación con el marido) y datos sobre la enfermedad (tipo de infertilidad, causa de la infertilidad, años de infertilidad, tiempo de tratamiento de fertilidad, si han recibido o no técnicas de reproducción asistida y etapa del tratamiento).

#### Cuestionario de Cogniciones Irracionales de Paternidad (Irrational Parenthood Cognitions Questionnaire IPCQ-14, por sus Siglas en Inglés)

El IPCQ fue desarrollado por Fkkes y luego traducido al chino por Lvy et al.^(12)^. La escala utilizada en este estudio constaba de 14 ítems, que medían la cognición de la maternidad de las mujeres en edad fértil mediante un método de puntuación de Likert de 5 puntos (1–5). La puntuación total en la escala oscilaba entre 14 y 70, donde las puntuaciones más altas indicaban más probabilidades de tener un hijo. Cuarenta y dos representaba el nivel medio. El α de Cronbach para la escala fue de 0,870.

#### La Escala de Estigma de la Infertilidad (Infertility Stigma Scale ISS por su Sigla en Inglés)-27

La ISS fue desarrollada por Fu et al.^(17)^. La escala utilizada para medir el estigma de las pacientes con infertilidad contiene 4 dimensiones y 27 ítems: autodesprecio (7 ítems), aislamiento social (5 ítems), humillación por parte de la gente que las rodea (6 ítems) y humillación por parte de la familia (9 ítems). Cada ítem se puntuó en una escala Likert de 5 puntos (1–5 puntos). La puntuación total en la escala oscilaba entre 27 y 135. Las puntuaciones más altas significaban más estigma. Las puntuaciones de 27 a 63 se clasificaron como estigma bajo, de 64 a 100 como estigma moderado y de 101 a 135 como estigma grave. El α de Cronbach para la escala total y cada dimensión osciló entre 0,77 y 0,94.

## ANÁLISIS Y TRATAMIENTO DE DATOS

Los datos se analizaron utilizando los programas IBM SPSS Statistics 27,0 y Mplus 8,3, SPSS Statistics 27,0 para la descripción estadística y pruebas de chi-cuadrado para las comparaciones de grupos. La regresión logística y la prueba H de Kruskal-Wallis evaluaron las relaciones entre variables. El software Mplus 8,3 se utilizó para el LPA. Los índices de ajuste del modelo de perfil potencial son: (1) Criterio de información de Akaike (AIC), Criterio de información bayesiano (BIC) y Criterio de información bayesiano ajustado (aBIC): cuanto más pequeños sean los valores de los tres indicadores, mejor será el ajuste del modelo.(2) La entropía representa la precisión de la clasificación, el rango de valores es de 0 a 1, cuanto mayor sea el valor de entropía, mayor será la precisión. (3) El valor P se probó basándose en la prueba de verosimilitud de Lo-Mendell Rubin (LMR) y la prueba de cociente de verosimilitud bootstrap (BLRT). *Un valor P* < 0,05 indica que el modelo es significativamente mejor que el modelo anterior.

## ASPECTOS ÉTICOS

Este estudio fue aprobado por el Comité de Ética del Hospital General de la Universidad Médica de Ningxia (KYLL-2024-1027). Todas las participantes firmaron el formulario de consentimiento informado (FCI) y participaron voluntariamente en este estudio.

## RESULTADOS

### Información General y Percepciones de Fertilidad Irracional y Puntuaciones de Estigma de las Encuestadas

En esta encuesta se distribuyeron un total de 302 cuestionarios, excluyendo 6 cuestionarios por la misma entrada de respuesta y orden de respuestas, se recuperaron 296 cuestionarios válidos y la tasa efectiva de recuperación de cuestionarios fue del 98,01%.

Un total de 296 mujeres infértiles de entre 20 y 48 años (31,34 ± 4,70) participaron en la encuesta, y el resto de la información general se muestra en la Tabla 1. La puntuación total de la CIP en pacientes femeninas infértiles osciló entre 14 y 70 (38,45 ± 13,30); la puntuación total del ISS osciló entre 27 y 135 (55,37 ± 21,89), la puntuación de la dimensión de autodesprecio osciló entre 7 y 35 (14,11 ± 6,40), la puntuación de la dimensión de aislamiento social fue de 5 a 25 (12,78 ± 5,18), la puntuación de la dimensión de estigma de las personas del entorno fue de 9 a 45 (17,23 ± 7,85), y la puntuación de la dimensión de estigma de la familia fue de 6 a 30 (11,25 ± 5,13).

**Tabla 1:** Análisis univariante del perfil latente de cognición de paternidad irracional en pacientes con infertilidad femenina – Yinchuan, Ningxia, China, 2023.

Variables		Total (*n* = 296)	Perfil 1 (*n* = 57)	Perfil 2 (*n* = 61)	Perfil 3(*n* = 76)	Perfil 4(*n* = 102)	E.S.	P
Edad	≤30	147 (49,7)	35 (61,4)	24 (39,3)	32 (42,1)	56 (54,9)	14,281	0,025
	31~	131 (44,3)	16 (28,1)	34 (55,7)	41 (53,9)	40 (39,2)		
	≥40	18 (6,1)	6 (10,5)	3 (4,9)	3 (3,9)	6 (5,9)		
Grupo étnico	Han	212 (72,0)	41 (71,9)	35 (57,4)	56 (73,7)	80 (78,4)	8,579	0,035
	Otros	84 (28,0)	16 (28,1)	26 (42,6)	20 (26,3)	22 (21,6)		
Estado civil	Primer casamiento	257 (86,8)	42 (73,7)	53 (86,9)	64 (84,2)	98 (96,1)	16,693	<0,001
	Nuevo casamiento	39 (13,2)	15 (26,3)	8 (13,1)	12 (15,8)	4 (3,9)		
Residencia	Rural	50 (16,9)	15 (26,3)	10 (16,4)	13 (17,1)	12 (11,8)	5,529	0,137
	Urbana	246 (83,1)	42 (73,7)	51 (83,6)	63 (82,9)	90 (88,2)		
Nivel educativo	Hasta secundaria completa	100 (33,8)	27 (47,4)	21 (34,4)	27 (35,5)	25 (24,5)	8,738	0,033
	Universitaria o más	196 (66,2)	30 (52,6)	40 (65,6)	49 (64,5)	77 (75,5)		
Ocupación	Desocupado	51 (17,2)	17 (29,8)	12 (19,7)	10 (13,2)	12 (11,8)	20,246	0,063
	Migrante	26 (8,8)	5 (8,8)	6 (9,8)	5 (6,6)	10 (9,8)		
	Empleado de empresas o instituciones	135 (45,6)	18 (31,6)	28 (45,9)	34 (44,7)	55 (53,9)		
	Independiente	41 (13,9)	6 (10,5)	7 (11,5)	11 (14,5)	17 (16,7)		
	Otros	43 (14,5)	11 (19,3)	8 (13,1)	16 (21,1)	8 (7,8)		
Ingreso familiar per cápita, yuan	≤3000	36 (12,2)	10 (17,5)	6 (9,8)	9 (11,8)	11 (10,8)	13,756	0,131
	3001~	131 (44,3)	30 (52,6)	34 (55,7)	31 (40,8)	36 (35,3)		
	5001~	84 (28,4)	12 (21,1)	12 (19,7)	23 (30,3)	37 (36,3)		
	≥8000	45 (15,2)	5 (8,8)	9 (14,8)	13 (17,1)	18 (17,6)		
Tipo de familia	Familia nuclear	242 (81,8)	44 (77,2)	50 (82,0)	66 (86,8)	82 (80,4)	–	0,540^(1)^
	Familia conyugal	6 (2,0)	0 (0,0)	1 (1,6)	2 (2,6)	3 (2,9)		
	Familia ampliada	6 (2,0)	3 (5,3)	0 (0,0)	1 (1,3)	2 (2,0)		
	Otros	42 (14,2)	10 (17,5)	10 (16,4)	7 (9,2)	15 (14,7)		
Tiempo de casamiento (años)	≤3	130 (43,9)	26 (45,6)	25 (41,0)	33 (43,4)	46 (45,1)	0,728	0,994
	4~7	69 (23,3)	14 (24,6)	14 (23,0)	17 (22,4)	24 (23,5)		
	≥7	97 (32,8)	17 (29,8)	22 (36,1)	26 (34,2)	32 (31,4)		
Hijos biológicos	NO	241 (81,4)	47 (82,5)	51 (83,6)	63 (82,9)	80 (78,4)	0,945	0,815
	SI	55 (18,6)	10 (17,5)	10 (16,4)	13 (17,1)	22 (21,6)		
Tipo de infertilidad	Primaria	166 (56,1)	28 (49,1)	37 (60,7)	43 (56,6)	58 (56,9)	1,672	0,645
	Secundaria	130 (43,9)	29 (50,9)	24 (39,3)	33 (43,4)	44 (43,1)		
Causas de infertilidad	Femenina	126 (42,6)	31 (54,4)	16 (26,2)	39 (51,3)	40 (39,2)	14,974	0,092
	Masculina	41 (13,9)	8 (14,0)	9 (14,8)	8 (10,5)	16 (15,7)		
	Factores de ambos	55 (18,6)	6 (10,5)	17 (27,9)	13 (17,1)	19 (18,6)		
	Desconocida	74 (25,0)	12 (21,1)	19 (31,1)	16 (21,1)	27 (26,5)		
Años de infertildad	≤1	68 (23,0)	10 (17,5)	12 (19,7)	16 (21,1)	30 (29,4)	4,651	0,589
	2~	126 (42,6)	25 (43,9)	26 (42,6)	36 (47,4)	39 (38,2)		
	≥5	102 (34,5)	22 (38,6)	23 (37,7)	24 (31,6)	33 (32,4)		
Años de tratamiento de infertilidad	≤1	121 (41,0)	19 (33,3)	25 (41,0)	29 (38,7)	48 (47,1)	10,599	0,102
	2~	131 (44,4)	27 (47,4)	25 (41,0)	31 (41,3)	48 (47,1)		
	≥5	43 (14,6)	11 (19,3)	11 (18,0)	15 (20,0)	6 (5,9)		
Técnica de reproducción asistida	Si	175 (59,1)	39 (68,4)	41 (67,2)	46 (60,5)	49 (48,0)	8,938	0,030
	No	121 (40,9)	18 (31,6)	20 (32,8)	30 (39,5)	53 (52,0)		
Estado del tratamiento	Esperando por resultados diagnósticos	83 (28,0)	15 (26,3)	19 (31,1)	12 (15,8)	37 (36,3)	–	0,028^(1)^
	Terapia con medicamentos/inyecciones	47 (15,9)	7 (12,3)	6 (9,8)	18 (23,7)	16 (15,7)		
	Terapia inseminación artificial	9 (3,0)	0 (0,0)	2 (3,3)	4 (5,3)	3 (2,9)		
	Fertilización In-Vitro (IVF)	157 (53,0)	35 (61,4)	34 (55,7)	42 (55,3)	46 (45,1)		
Grado de estrés por infertilidad	Muy importante	63 (21,3)	23 (40,4)	14 (23,0)	16 (21,1)	10 (9,8)	–	<0,001^(1)^
	Relativamente importante	102 (34,5)	20 (35,1)	28 (45,9)	21 (27,6)	33 (32,4)		
	Normal	114 (38,5)	12 (21,1)	17 (27,9)	34 (44,7)	51 (50,0)		
	Relativamente menor	9 (3,0)	1 (1,8)	1 (1,6)	3 (3,9)	4 (3,9)		
	Muy menor	8 (2,7)	1 (1,8)	1 (1,6)	2 (2,6)	4 (3,9)		
Relación con el marido	Muy buena	183 (61,8)	33 (57,9)	37 (60,7)	45 (59,2)	68 (66,7)	–	0,261^(1)^
	Relativamente buena	100 (33,8)	18 (31,6)	21 (34,3)	29 (38,2)	32 (31,4)		
	Normal	12 (4,1)	6 (10,5)	2 (3,3)	2 (2,6)	2 (2,0)		
	Muy mala	1 (0,3)	0 (0,0)	1 (1,6)	0 (0,0)	0 (0,0)		
Escala de Estigma de Infertilidad (Infertility Stigma Scale) M(P25, P75)	Auto-desprecio	78,00 (62,00,96,50)	22,00 (12,00,27,00)	18,00 (12,00,21,00)	25,00 (18,00,35,00)	16,00 (13,00,19,00)	83,724	<0,001
	Retiro de la sociedad	61,00 (51,50,71,00)	16,00 (11,50,18,50)	14,00 (11,50,18,00)	18,00 (12,00,22,00)	12,00 (7,00,15,00)	82,986	<0,001
	Humillación por el grupo alrededor	54,00 (38,50,61,00)	13,00 (10,00,15,00)	12,50 (10,00,17,00)	17,00 (10,00,19,00)	12,00 (6,00,13,00)	90,716	<0,001
	Humillación por la familia	38,00 (30,00,54,00)	10,00 (7,00,14,00)	10,00 (6,00,12,00)	9,00 (9,00,18,00)	6,00 (6,00,12,00)	89,252	<0,001

(1) Método de probabilidad exacta de Fisher. Perfil 1: grupo de CIP alto; Perfil 2: grupo de expectativas de CIP medio; Perfil 3: grupo de baja autoestima de CIP medio; Perfil 4: grupo de CIP bajo.

#### Perfil Latente de Cognición de Paternidad Irracional en Pacientes Femeninas Infértiles

En este estudio se ajustaron un total de 1-5 modelos, como se muestra en la Tabla 2. Los valores más bajos de AIC, BIC y aBIC indican una mayor precisión. El valor de entropía es > 0,9 para los números de clase 2, 3, 4 y 5, pero el LMR para las clases 3 y 5 no alcanza significación estadística (*P* > 0,05). Las probabilidades atribuidas a los cuatro perfiles latentes fueron 0,950, 0,959, 0,980 y 0,977, respectivamente, todas ellas > 90,00%, lo que indica que el modelo de los cuatro perfiles latentes era creíble. Por lo tanto, se seleccionaron cuatro perfiles latentes para el análisis. De acuerdo con los resultados de la clasificación, se dibujó el diagrama de probabilidad condicional del perfil latente, como se muestra en la Figura 1. Había 57 casos (19,30%) en el grupo Perfil 1, y la puntuación media de cada ítem estaba en un nivel alto, lo que se denominó «grupo CIP alto». Tanto el grupo Perfil 2 como el Perfil 3 se situaron en el medio, por lo que ambos se denominaron «CIP moderado». Hubo 61 casos (20,60%) en el grupo Perfil 2, que obtuvo puntuaciones medias más altas en el ítem 1 «Tener un hijo es lo más importante en la vida», el ítem 2 «vivir sin un hijo es inútil y vacío», el ítem 13 «No tener un hijo es un tormento de por vida» y el ítem 14 «Una persona haría cualquier cosa por tener un hijo». Por lo tanto, se denominó «CIP medio - grupo de expectativas»; había 76 casos en el grupo Perfil 3 (25,70%), y este grupo tenía puntuaciones medias más altas en el ítem 3, «Algunas personas tienen hijos fácilmente, a otras les resulta difícil tener hijos, lo cual es ridículo», el ítem 7, «Cuando no puedes tener hijos, te sientes inferior a los demás», y el ítem 8, «Cuando no puedes tener hijos, empiezas a odiar tu cuerpo». Por lo tanto, se denominó «grupo de puntuación de inteligencia cognitiva media - baja autoestima». Hubo 102 casos (34,50%) en el grupo de perfil 4, y la puntuación media de cada ítem fue baja, lo que se denominó «grupo de puntuación de inteligencia cognitiva baja».

**Tabla 2:** Indicadores de ajuste del análisis de perfiles latentes de cognición de paternidad irracional en pacientes femeninas infértiles – Yinchuan, Ningxia, China, 2023.

Modelo	AIC	BIC	aBIC	Entropía	LMR	BLRT	Probabilidad de clase (%)
Perfil 1	14420,301	14523,631	–	–	–	–	100
Perfil 2	13081,285	13239,970	13103,603	0,926	<0,001	<0,001	48,986/51,014
Perfil 3	12594,068	12808,109	12624,172	0,933	0,2374	<0,001	37,838/41,554/20,608
**Perfil 4**	**12424,995**	**12694,392**	**12462,885**	**0,936**	**0,0186**	**<0,001**	**25,676/20,608/34,459/19,257**
Perfil 5	12301,187	12625,939	12346,863	0,929	0,5054	<0,001	28,041/18,243/14,189/20,270/19,257

**Figura 1:** Perfiles latentes de cognición irracional de la paternidad en pacientes femeninas con infertilidad – Yinchuan, Ningxia, China, 2023.

#### Análisis de Varianza Unidireccional del Perfil Latente de Cognición Irracional de la Fertilidad en Pacientes Femeninas Infértiles

El perfil latente de la CIP de las pacientes femeninas infértiles se comparó en cuanto a edad, etnia, estado civil, nivel educativo, recepción de tecnología de reproducción asistida, etapa de tratamiento y presión de fertilidad, y las diferencias fueron estadísticamente significativas (*p* < 0,05), como se muestra en la Tabla 1.

#### Análisis de Regresión Logística Multinomial del Perfil Latente de Cognición Irracional de la Paternidad en Pacientes Femeninas Infértiles

Para el análisis se utilizó el comando R3STEP del método robusto de tres pasos. Se realizó un análisis de regresión logística multinomial con los cuatro perfiles latentes como variables dependientes y las variables que fueron estadísticamente significativas en el análisis univariante como variables independientes. A las variables dependientes se les asignaron los siguientes valores: grupo de IPC alto = 1; grupo de IPC medio – expectativa = 2; grupo de IPC medio – baja autoestima = 3; y grupo de IPC bajo (grupo de referencia). Las variables independientes se asignaron de la siguiente manera: edad: ≤30 (1, 0), 31~(0, 1), ≥40 (0, 0); grupo étnico Han = 1, otro grupo étnico = 2; estado civil: primer matrimonio = 1, segundas nupcias = 2; nivel educativo: Postsecundaria e inferior = 1, Licenciatura o superior = 2; tecnología de reproducción asistida: sí = 1, no = 2; etapa de tratamiento: en espera de resultados diagnósticos (1, 0, 0), terapia con medicamentos/inyecciones (0, 1, 0), terapia de inseminación artificial (0, 0, 1), FIV (0, 0, 0); grado de estrés por fertilidad: muy alto (1, 0, 0, 0), relativamente alto (0, 1, 0, 0), general (0, 0, 1, 0), relativamente bajo (0, 0, 0, 1), muy bajo (0, 0, 0, 0). Los resultados mostraron que las pacientes infértiles de etnia Han y en primeros matrimonios tenían un grupo de CIP bajo, y aquellas que recibieron tecnología de reproducción asistida tenían una CIP alto y medio y esperaban tener un hijo. Véase la Tabla 3.

**Tabla 3:** Análisis de regresión logística del perfil potencial de cognición irracional de paternidad en pacientes femeninas con infertilidad – Yinchuan, Ningxia, China, 2023.

Factores influyentes	Variables	Perfil 1			Perfil 2			Perfil 3		
		*OR*值 (95%*CI*)	*B*	*P*值	*OR*值 (95%*CI*)	*B*	*P*值	*OR*值 (95%*CI*)	*B*	*P*值
Age	≤30	1,158 (0,307–7,508)	0,418	0,609	0,978 (0,198–4,834)	−0,022	0,978	1,380 (0,295–6,450)	0,322	0,682
	31~	0,261 (0,050–1,366)	−1,343	0,112	1,301 (0,267–6,348)	0,263	0,745	2,016 (0,434–9,351)	0,701	0,371
Grupo étnico	Han	0,794 (0,340–1,854)	−0,230	0,595	0,358 (0,172–0,745)	−1,028	0,006	0,725 (0,349–1,505)	−0,322	0,388
Estado civil	Primer casamiento	0,053 (0,012–0,224)	−2,947	<0,001	0,275 (0,066–1,149)	−1,293	0,077	0,279 (0,076–1,023)	−1,278	0,054
	Nivel educativo	Hasta secundaria completa	1,757 (0,761–4,057)	0,564	0,187	1,165 (0,542–2,505)	0,153	0,695	1,198 (0,588–2,438)	0,108	0,619
	Técnica de reproducción asistida	Si	3,396 (1,082–10,658)	1,223	0,036	3,650 (1,266–10,525)	1,295	0,017	1,733 (0,635–4,731)	0,550	0,283
Estado de tratamiento	Esperando por resultados diagnósticos	1,895 (0,573–6,264)	0,639	0,295	2,196 (0,731–6,596)	0,786	0,161	0,600 (0,196–1,838)	−0,051	0,371
	Terapia con medicamentos/inyecciones	1,962 (0,478–8,053)	0,674	0,349	1,641 (0,426–6,323)	0,495	0,472	2,274 (0,716–7,221)	0,821	0,164
	Terapia inseminación artificial	–	−20,043	–	1,079 (0,140–8,329)	0,076	0,942	1,942 (0,361–10,451)	0,664	0,440
Realación con el marido	Muy buena	10,716 (0,938–122,456)	2,372	0,056	4,920 (0,422–57,345)	1,593	0,203	3,836 (0,492–29,914)	1,344	0,200
	Relativamente buena	3,226 (0,303–34,630)	1,171	0,332	2,733 (0,26–28,694)	1,006	0,402	1,305 (0,187–9,130)	0,266	0,788
	Normal	0,743 (0,069–8,004)	−0,297	0,807	1,282 (0,121–13,595)	0,248	0,837	1,693 (0,248–11,540)	0,526	0,591
	Muy mala	1,011 (0,039–25,919)	0,011	0,995	0,984 (0,039–24,802)	−0,016	0,992	1,385 (0,122–15,747)	0,326	0,793

Las variables independientes fueron edad ≥40 años, otros grupos étnicos, segundo casamiento, universitario o superior, ninguna tecnología de reproducción asistida, IVF y estrés de fertilidad muy pequeño como grupo de referencia.

#### Relación Entre la Cognición Irracional de la Paternidad y el Estigma Patológico en Pacientes Infértiles de Sexo Femenino

La comparación de las puntuaciones totales del ISS y las puntuaciones de cada dimensión entre pacientes infértiles de sexo femenino con cuatro perfiles latentes de CIP mostró diferencias estadísticamente significativas (*P* < 0,05), como se muestra en la Tabla 1. Tras controlar los factores de confusión de edad, etnia, estado civil, nivel educativo, aceptación de la tecnología de reproducción asistida, fase de tratamiento y estrés de fertilidad, se realizó un análisis de regresión lineal múltiple con la puntuación total del ISS como variable dependiente y los cuatro perfiles latentes de CIP como variable independiente. Los resultados mostraron que la CIP alto y la CIP medio - expectativas y la CIP medio - baja autoestima predecían positivamente el estigma en comparación con la CIP bajo (todos *P* <0,05), explicando el 44,2% de la varianza total. Ver Tabla 4.

**Tabla 4:** Análisis de regresión lineal múltiple del perfil latente percibido de paternidad irracional y estigma en pacientes femeninas con infertilidad – Yinchuan, Ningxia, China, 2023.

Perfil	*B*	*SE*	*t*	*P*	95%*CI*
Constante	57,445	9,418	6,099	<0,001	38,906~75,983
Grupo de alta cognición irracional de la paternidad	34,628	2,953	11,727	<0,001	28,816~40,440
Grupo de mediana expectativa de cognición irracional de la paternidad	16,256	2,769	5,870	<0,001	10,805~21,707
Grupo de mediana expectativa de cognición irracional de la paternidad con baja autoestima	10,249	2,528	4,054	<0,001	5,273~15,226

*R* ^2^ = 0,461, adjusted *R* ^2^ = 0,442, F = 24,405, *P* < 0,001.

## DISCUSIÓN

### Existe Heterogeneidad en la Cognición Irracional de la Paternidad de las Pacientes Infértiles

Las mujeres infértiles muestran heterogeneidad en su CIP. Utilizando LPA, identificamos 4 perfiles distintos de CIP entre las mujeres infértiles. El grupo de CIP más alto (Perfil 1), el grupo de CIP medio – expectativas (Perfil 2), el grupo de CIP medio – baja autoestima (Perfil 3), y el grupo de CIP más bajo (Perfil 4). El grupo de CIP alto (Perfil 1) incluyó 57 casos (19,30%), lo que indica un fuerte deseo de alcanzar una vida feliz mediante la maternidad. Esto sugiere que los profesionales sanitarios deben trasladar las intenciones de fertilidad de las pacientes y orientar correctamente los conceptos de fertilidad para evitar una CIP excesiva durante el tratamiento de la infertilidad. Los perfiles 2 y 3, que representaban el 46,30% de la muestra, presentaban niveles medios de CIP, pero diferían en sus características subyacentes. Por lo tanto, el presente estudio utilizó el LPA para identificar la heterogeneidad de la CIP en dichos pacientes. La identificación precoz y las intervenciones cognitivas a medida son cruciales para los individuos del perfil 1. El perfil 4, el grupo más numeroso (34,50%), mostró una CIP más bajo, lo que sugiere la necesidad de explorar las motivaciones intrínsecas para la fertilidad y mejorar su perspectiva cognitiva.

### Análisis de los Factores que Influyen en un Perfil Latente de Cognición Irracional de la Paternidad en Pacientes Femeninas Infértiles

Los resultados de este estudio mostraron que la CIP era inferior en las pacientes con infertilidad que se habían casado por primera vez, en comparación con las que se habían vuelto a casar (*OR* = 0,053, *P* < 0,001). El estado civil influye en las intenciones de fertilidad, y las personas primogénitas presentan niveles más bajos de CIP^(18)^. Los pacientes primogénitos acaban de contraer matrimonio y tienen un estado civil estable, por lo que tienen una menor disposición a tener hijos y una CIP más bajo. Por lo tanto, en el caso de las pacientes primogénitas, la infertilidad debe tratarse activamente, deben establecerse conceptos científicos de fertilidad y debe mejorarse la CIP. Además, en este estudio también se observó que, en comparación con las minorías étnicas, las pacientes de etnia Han con infertilidad tenían una CIP más bajo (*OR* = 0,358; *P* = 0,006), similar a los resultados de Shreffler^(19)^. La intención de fertilidad en sí es significativamente específica de cada cultura, y las diferencias étnicas son un aspecto importante^(20)^. Ningxia pertenece a las zonas de minorías étnicas y, debido a las ideas culturales tradicionales, las minorías étnicas se casan más temprano y tienen embarazos más precoces, mientras que la etnia Han se casa más tarde y tiene embarazos más tardíos, lo que se traduce en una CIP más bajo. Por lo tanto, las ideas tradicionales sobre la fertilidad deberían modificarse para corregir el sesgo dla CIP en respuesta a las diferencias culturales.

Los resultados de este estudio mostraron que las pacientes con infertilidad que recibieron tecnología de reproducción asistida tenían un IPC más alto (*OR* = 3,396; *P* = 0,036) o moderado (*OR* = 3,650; *P* = 0,017) en comparación con las que no recibieron tecnología de reproducción asistida, resultados similares a los de un estudio extranjero^(21)^. La naturaleza exigente de la tecnología de reproducción asistida, incluidos los procedimientos invasivos, los costes elevados y el estrés emocional, puede contribuir a aumentar la CIP. La infertilidad como experiencia estigmatizante y aislante es un factor de estrés crónico^(22)^. Durante la concepción asistida, los tratamientos largos y agotadores, los elevados costes médicos, los procedimientos invasivos, las presiones de la familia y la sociedad y, especialmente, el fracaso de la concepción asistida pueden causar diversos grados de estrés fisiológico y psicológico en los pacientes infértiles, lo que puede dar lugar a una CIP mayor y moderado. Por lo tanto, debemos centrarnos en la CIP de los pacientes infértiles con tecnología de reproducción asistida, guiar activamente el concepto correcto de la cognición de la fertilidad, popularizar el conocimiento de la visión racional de la cognición de la fertilidad y mejorar el nivel de CIP.

### Relación Entre las Características Cognitivas Irracionales de la Paternidad y el Estigma en Mujeres Infértiles

Los resultados de este estudio mostraron que el grupo de CIP más alto tenía las puntuaciones más altas en las cuatro dimensiones del estigma de la infertilidad, autodesprecio, retraimiento social, humillación por parte de la multitud circundante y humillación por parte de la familia, seguido por el grupo de CIP medio – expectativas, el grupo de CIP medio – baja autoestima y, por último, el grupo de CIP más bajo. Un mayor CIP se asocia a un mayor deseo de tener un hijo, a la autoculpabilidad y a la insatisfacción con la infertilidad, así como a un mayor miedo al juicio social, a evitar los temas relacionados con los hijos y a una mayor sensibilidad a los estímulos relacionados con los hijos. El grupo de CIP medio – expectativas y el grupo de CIP medio – baja autoestima tienen las puntuaciones medias de las cuatro dimensiones, lo que indica que su deseo de tener un hijo se sitúa en un nivel medio. El deseo aparente de ambos perfiles de tener un hijo era medio, pero sus características subyacentes diferían. Por el contrario, el grupo de CIP bajo obtuvo la puntuación más baja de las 4 dimensiones, lo que indica que los pacientes no deseaban especialmente tener un hijo. En consecuencia, tampoco se preocupan por lo que piensen ellos y los demás, y no se producen una serie de cambios psicológicos como sentimientos de vergüenza.

Además, este estudio también descubrió que, tras controlar los factores de confusión, la CIP más alto, la CIP medio – expectativa y la CIP medio - baja autoestima predecían positivamente el estigma por enfermedad en comparación con la CIP más bajo (todos *P* < 0,05). La vergüenza por la enfermedad, o estigma, implica sentimientos de diferencia, rechazo e inaceptación debido a la enfermedad, que pueden conducir a la estigmatización y la autodesvalorización durante los episodios de infertilidad, y está estrechamente relacionada con el estrés por fertilidad en pacientes con infertilidad^(23)^. El estigma, como emoción negativa, puede provocar alteraciones en la secreción de hormonas sexuales, lo que puede afectar a la fertilidad y reducir la tasa de éxito de la concepción asistida^(24)^. La CIP, en particular las altas expectativas, y la baja autoestima pueden exacerbar los sentimientos de enfermedad y vergüenza, lo que conduce a un aumento del estrés relacionado con la fertilidad^(25)^. En la clínica, la CIP puede utilizarse como punto de entrada para la intervención, y mediante intervenciones psicológicas como la terapia de pensamiento positivo, la terapia cognitiva de grupo y la terapia racional emotiva conductual, podemos ayudar a los pacientes a sustituir los estilos de pensamiento y las creencias irracionales por estilos de pensamiento y creencias racionales, para reducir la aparición de la vergüenza por la enfermedad.

## CONCLUSIÓN

En este estudio, la CIP de pacientes femeninas con infertilidad fue categorizado en cuatro perfiles por LPA: grupo de CIP alto, CIP medio – grupo de expectativas, CIP medio – grupo de baja autoestima, y grupo de CIP bajo. Existían características significativas entre el perfil latente y estaban influidas por el estado civil, la etnia y si la tecnología de reproducción asistida era o no un factor. Además, este estudio muestra ahora que los rasgos de CIP en mujeres infértiles pueden predecir positivamente el estigma. Por lo tanto, el personal sanitario debería identificar las características de las diferentes categorías de pacientes en una fase temprana, utilizar la CIP como punto de entrada para la intervención y movilizar activamente todos los aspectos del apoyo social para establecer una visión correcta de la cognición de la paternidad en los pacientes y reducir el nivel de estigma en los pacientes con infertilidad.
